# Rich topological nodal line bulk states together with drum-head-like surface states in NaAlGe with *anti*-PbFCl type structure

**DOI:** 10.1016/j.jare.2020.01.017

**Published:** 2020-01-31

**Authors:** Xiaotian Wang, Guangqian Ding, Zhenxiang Cheng, Gokhan Surucu, Xiao-Lin Wang, Tie Yang

**Affiliations:** a Institute for Superconducting and Electronic Materials (ISEM), University of Wollongong, Wollongong 2500, Australia; b School of Science, Chongqing University of Posts and Telecommunications, Chongqing 400065, China; c Department of Physics, Middle East Technical University, Turkey; d Department of Electric and Energy, Ahi Evran University, Turkey; e ARC Centre of Excellence in Future Low Energy Electronics Technologies (FLEET), University of Wollongong, Wollongong, NSW 2500, Australia; f School of Physical Science and Technology, Southwest University, Chongqing 400715, China

**Keywords:** Surface states, *Anti*-PbFCl type, Electronic structures, First-principles, TNL states

## Abstract

The band topology in condensed matter has attracted widespread attention in recent years. Due to the band inversion, topological nodal line semimetals (TNLSs) have band crossing points (BCPs) around the Fermi level, forming a nodal line. In this work, by means of first-principles, we observe that the synthesized NaAlGe intermetallic compound with *anti*-PbFCl type structure is a TNLS with four NLs in the *k*_z_ = 0 and *k*_z_ = *π* planes. All these NLs in NaAlGe exist around the Fermi level, and what is more, these NLs do not overlap with other bands. The exotic drum-head-like surface states can be clearly observed, and therefore, the surface characteristics of NaAlGe may more easily be detected by experiments. Biaxial strain has been explored for this system, and our results show that rich TNL states can be induced. Furthermore, the spin-orbit coupling effect has little effect on the band structure of NaAlGe. It is hoped that this unique band structure can soon be examined by experimental work and that its novel topological elements can be fully explored for electronic devices.

## Graphical abstract

**Figure:**
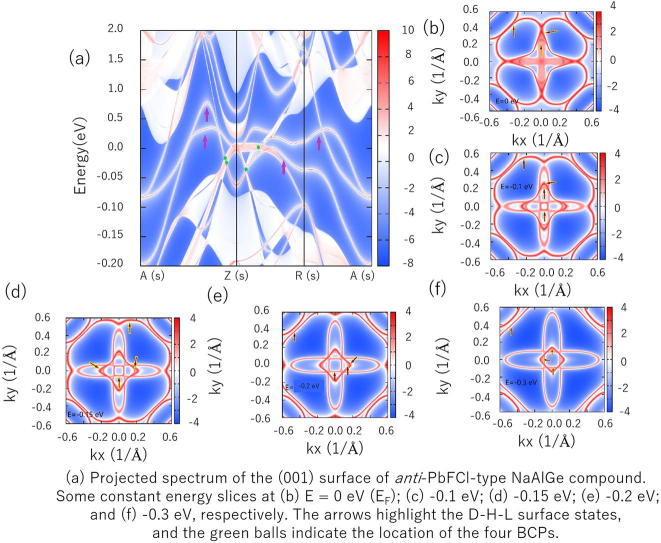

## Introduction

Topological insulators [1], [2], [3], [4], [5] have been the hotspot of modern condensed matter physics for several years. The main features of topological insulators can be expressed as follows: (1) they exhibit energy band inversion and a bulk band gap caused by strong spin-orbit coupling; (2) they possess gapless boundary states, i.e., their surface states have metallic properties. Different types and families of topological insulators have been widely investigated since the discovery of topological insulators in HgTe/CdTe quantum wells [6]. More interestingly, Miao et al. [7] found that nanoscale engineering can convert conventional semiconductors (with a sizable band gap and small spin-orbit coupling effect) into topological insulators. This research opens up new routes for designing topological insulator candidate materials.

Recently, another class of materials containing interesting topological elements, namely, topological semimetals (TSMs) [8], [9], [10], [11], [12], [13], [14], [15], [16], [17], [18], [19], [20], [21], [22], [23], [24], [25], has received wide attention. Compared with topological insulators, TSMs have a special topological surface state, interesting magnetic transport properties, and extremely high carrier mobility. TSMs are characterized by non-trivial band crossings (owing to the band inversion) between the conduction band and the valence band in the momentum space. Around the band crossings, the quasiparticles behave differently from the usual Schrödinger type fermions. According to the degeneracy of band crossing points (BCPs) and their distribution in the Brillouin zone, TSMs can be classified into the following types: Dirac semimetals [26], [27], [28], Weyl semimetals [29], [30], and topological nodal line semimetals (TNLS) [31]. A Dirac semimetal has two bands with double degeneracy, and the two bands cross each other at or along high symmetry points near the Fermi level (*E*_F_). On the other hand, its band crossings also receive protection from the crystal symmetry. In a Weyl semimetal, two non-degenerate band crossings can be observed around the *E*_F_. Moreover, no crystal symmetry protection is required for a Weyl semimetal. Significantly different from the isolated points in the Dirac and Weyl semimetals, for a TNLS, the crossings between the bands can form one-dimensional (1D) nodal lines (NLs)/loops in three-dimensional (3D) momentum space under certain crystal symmetries. Depending on the slope of the energy band dispersion in the momentum-energy space, TSMs can be viewed as two types [32], [33]: type I and type II TSMs. For type I TSMs, these bands exhibit a traditional conical dispersion in which the electron and hole regions are well separated by energy. For type II TSMs, these bands are fully tilted, and their electron and hole states coexist at a given energy. There is also the possibility, however, that the NLs in the TSMs are composed of type I and type II crossing points (CPs), and this new type of TSM is denoted as the hybrid type [34]. The physical properties of type I, type II, and hybrid type TSMs are quite different [35], [36].

In this work, we focus on an experimentally synthesized intermetallic compound, NaAlGe [37] with an *anti*-PbFCl-type lattice structure. We theoretically prove that NaAlGe hosts TNL states near the *E*_F_. What is more, this material exhibits the following advantages: (1) There are no other external energy bands near the TNLs; (2) The energy band crossing produces a total of four NLs, so the signal of the expected NLs would be very obvious for experimental detection. Finally, biaxial strain was applied on this material and successfully induced different TNL state transitions in NaAlGe compound.

## Materials and methods

The crystal structures have been totally relaxed in this work (see Fig. S1) with the help of density functional theory (DFT), and the obtained theoretical lattice parameters are *a*/*b* = 4.189 Å and *c* = 7.414 Å. The theoretical lattice constants that we obtained are consistent with the experimental values [37], i.e. *a*/*b* = 4.164 Å and *c* = 7.427 Å. NaAlGe crystallizes in a tetragonal structure with the *P4/nmm* space group (No. 129). This unit cell contains six atoms, i.e., two Na atoms, two Al atoms, and two Ge atoms, respectively. The Na, Al, and Ge atoms occupy the (0.5, 0.0, 0.64), (0.0, 0.0, 0.0), and (0.5, 0.0, 0.21) Wyckoff sites, respectively. In this study, we calculated the band structure of NaAlGe using density functional theory, within the VASP code [38]. The Perdew-Burke-Ernzerhof (PBE) [39] parameterization of the generalized gradient approximation (GGA) [40] was selected to describe the exchange and correlation functionals. We also used the projector augmented wave (PAW) [41] method to deal with the interaction between the ion cores and valence electrons. For the *anti*-PbFCl type NaAlGe system, a plane-wave basis set cut-off of 500 eV and a Monkhorst-Pack special 13 × 13 × 7 k-point mesh were used in the Brillouin zone integration. The unit cell was optimized until the force and total energy were less than 0.005 eV/Å and 0.0000001 eV, respectively. The phonon energy calculation for NaAlGe was performed in NanoAcademic Device Calculator (Nanodcal) code [42]. As shown in Fig. S2, we utilized the phonon spectrum to test the stability of the tetragonal NaAlGe compound. The absence of a virtual frequency guarantees the stability of the tetragonal state of NaAlGe. Therefore, we can conclude that the tetragonal NaAlGe is structurally stable. Also, the elastic constant and mechanical properties (see Tables S1 and S2) of NaAlGe compound have been studied, and the results are given in the Supplementary Information. The mechanical stability of this system was also evaluated based on the obtained elastic constant. The surface states of NaAlGe were investigated in this study via the WannierTools software package [43] according to the method of maximally localized Wannier functions [44], [45].

## Results and discussion

**Fig. 1:**
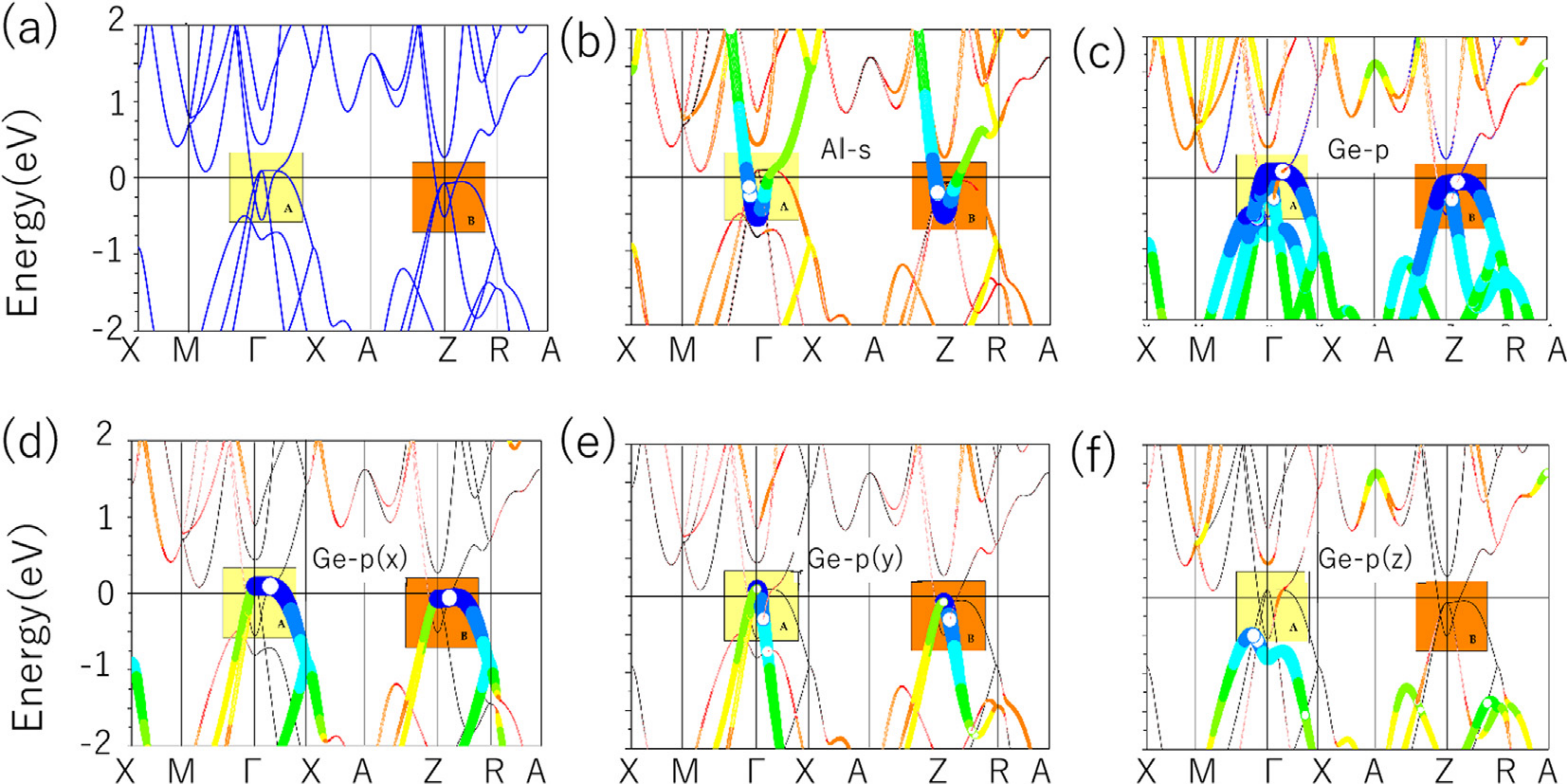
(a) Band structure of *anti*-PbFCl-type NaAlGe calculated with the help of PBE along the high symmetry points X-M- Γ -X-A-Z-R-A in the bulk Brillouin zone; (b)-(f) Orbital-resolved band structures of *anti*-PbFCl-type NaAlGe calculated with the help of PBE.

Fig. 1(a) exhibits the band structures of *anti*-PbFCl-type NaAlGe that were calculated with the help of PBE along the high symmetry points X-M-Γ-X-A-Z-R-A in the bulk Brillouin zone (see Fig. S3). In this figure, we do not consider the effect of spin-orbit coupling (SOC) due to the fact that Na, Al, and Ge are not heavy elements. We will also discuss the influence of the SOC on the band structures later in this manuscript. From Fig. 1(a), one can see that the NaAlGe system exhibits metallic properties due to the bands and the Fermi level overlapped with each others [46]. Furthermore, one can see that there are some BCPs near the *E*_F_ (range from −0.4 eV to 0 eV). We can see that the band crossing points are mainly concentrated in two regions, marked as A and B. In order to make our results more accurate, we repeated the calculation of the band structures of NaAlGe using the state-of-the-art Heyd-Scuseria-Ernzerhof (HSE06) [47], [48] functional, and the results are shown in Fig. S4(a). By comparing the results of PBE and HSE06, we found that the band structures near the *E*_F_ are basically the same. That is to say, the inverse band topology [49] can be clearly found near the E_F_ and the BCPs occurred in regions A and B.

**Fig. 2:**
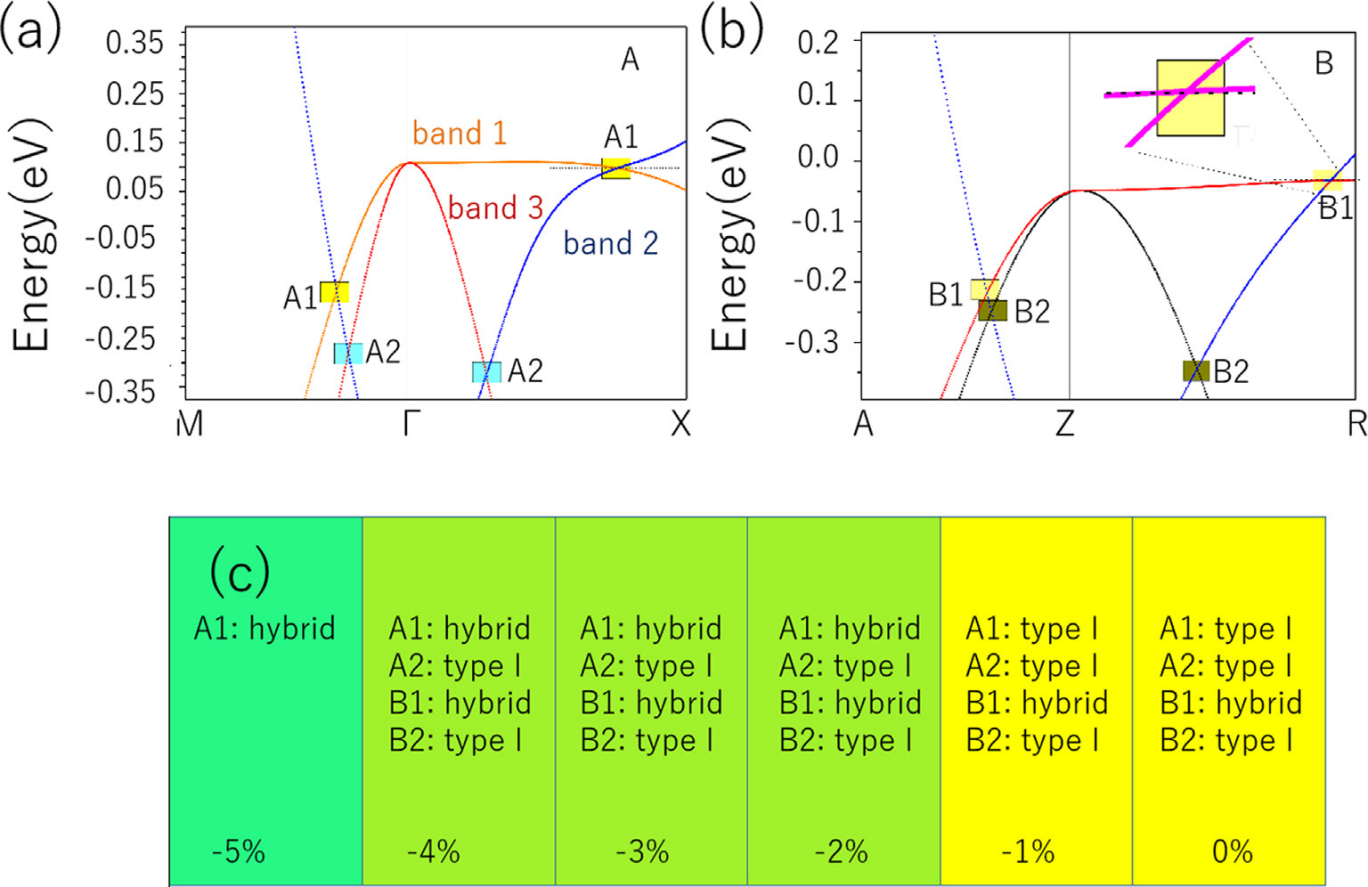
(a) and (b) Band structure of *anti*-PbFCl-type NaAlGe, calculated with the help of PBE along the M- Γ -X and A-Z-R directions, respectively, in the bulk Brillouin zone; (c) Possible TNL state transitions in NaAlGe under biaxial strain (−5% − 0%) in the *ab*-plane.

Next, we will discuss the two regions A and B, respectively. For region A, four BCPs along the M- Γ -X direction can be observed, and these four CPs arise from the crossings of three bands, i.e., bands 1, 2, and 3 (see Fig. 2(a)). In detail, there are two BCP A1 along the M- Γ -X path, one is the M- Γ and the other one is along the Γ -X path. Both A1 BCPs are arising from the intersection of band 1 (orange line) and band 2 (blue line). Similar to BCPs A1, two BCPs A2, which coming from the crossings of band 2 and band 3 (red line), are along M- Γ and Γ -X directions, respectively.

**Fig. 3:**
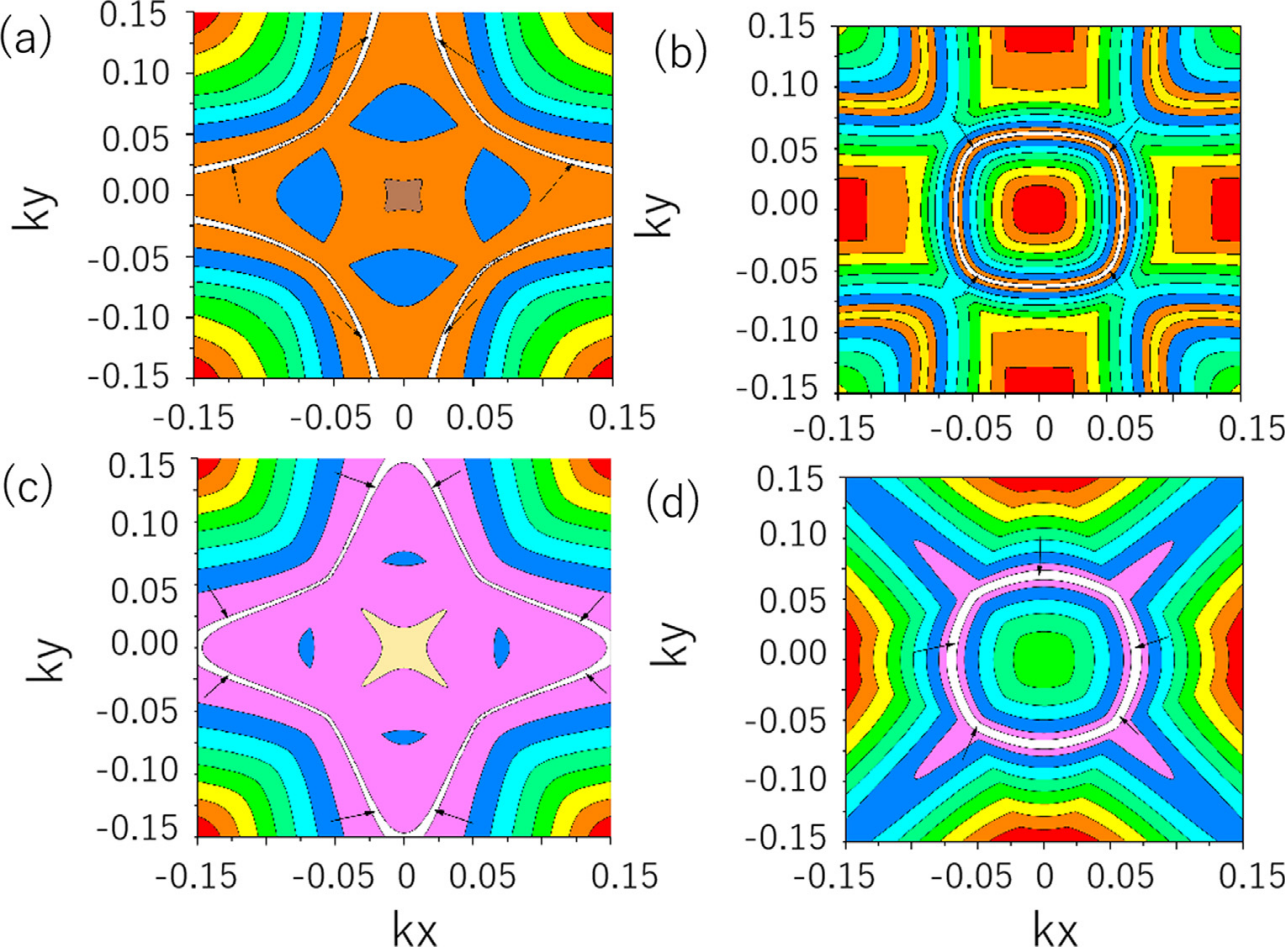
(a) and (b) Schematic diagram of the A1 and A2 TNLs in the *k*_z_ = 0 plane; (c) and (d) Schematic diagram of the B1 and B2 TNLs in the *k*_z_ = *π* plane. The TNLs are highlighted as white lines.

Based on the calculated orbital-resolved band structures [50] in Fig. 1(b)–(f), one can see that the band 1 in region A is coming from the Ge-*p(x)* orbitals, while band 2 is formed from Al-*s* orbitals, and band 3 is mainly arising from the Ge-*p(y)* orbitals. Therefore, the two A1 crossing points are formed by the hybridization between the Al-*s* and the Ge*-p(x)* orbitals; the two A2 crossing points are formed by the hybridization between the Al-*s* and the Ge*-p(y)* orbitals. As shown in Fig. 2(a), we can see that all of these four crossing points in region A have double degeneracy. Furthermore, for the NaAlGe system, the spin effect was not included because the nonmagnetic state is the most stable ground state for this system. Also, NaAlGe compound was protected from time reversal (*T*) symmetry and spatial inversion (*P*) symmetries. Therefore, we can conclude that such BCPs cannot be seen as isolated nodal points [51], [53] when the role of the SOC is not taken into account. As shown in Fig. 3(a) and (b), one can see that these four CPs belong to two NLs (A1 and A2) that are centered around the Γ point in the *k*_z_ = 0 plane. From Figs. 2(a), 3(a), and (b), we can see that TNL A1 has higher energy and larger size than TNL A2.

For region B, there are also four CPs along the A-Z-R direction. Since the energy bands of regions A and B are roughly the same, therefore, there are also two TNLs (named as B1 and B2) [53] centered around the Z point in the *k*_z_ = *π* plane. Two BCPs belong to NL B1, and two BCPs are parts of NL B2. Similar to region A, the schematic diagrams of these two NLs (B1 and B2) in the *k*_z_ = *π* plane are also given in Fig. 3(c) and (d), respectively.

Based on above-mentioned information, nodal points can be divided into two types, namely, type-I and type-II [32], [33], according to the slope of the energy band dispersion at these BCPs. As shown in Fig. 2(a), both nodal points of A1 along the M- Γ and Γ -X directions in region A are type I, and therefore, one can see that the A1 NL corresponds to type-I. The same situation can be seen in the A2 and B2 NLs. The nodal point of B1 along the A-Z direction in region B is type I, whereas the nodal point of B1 along the Z-R direction in this region is type II (see the inset figure in Fig. 2(b)), reflecting the fact that the TNL B1 contains both type -I and type II nodal points at the same time and is thus a hybrid type NL [34].

**Fig. 4:**
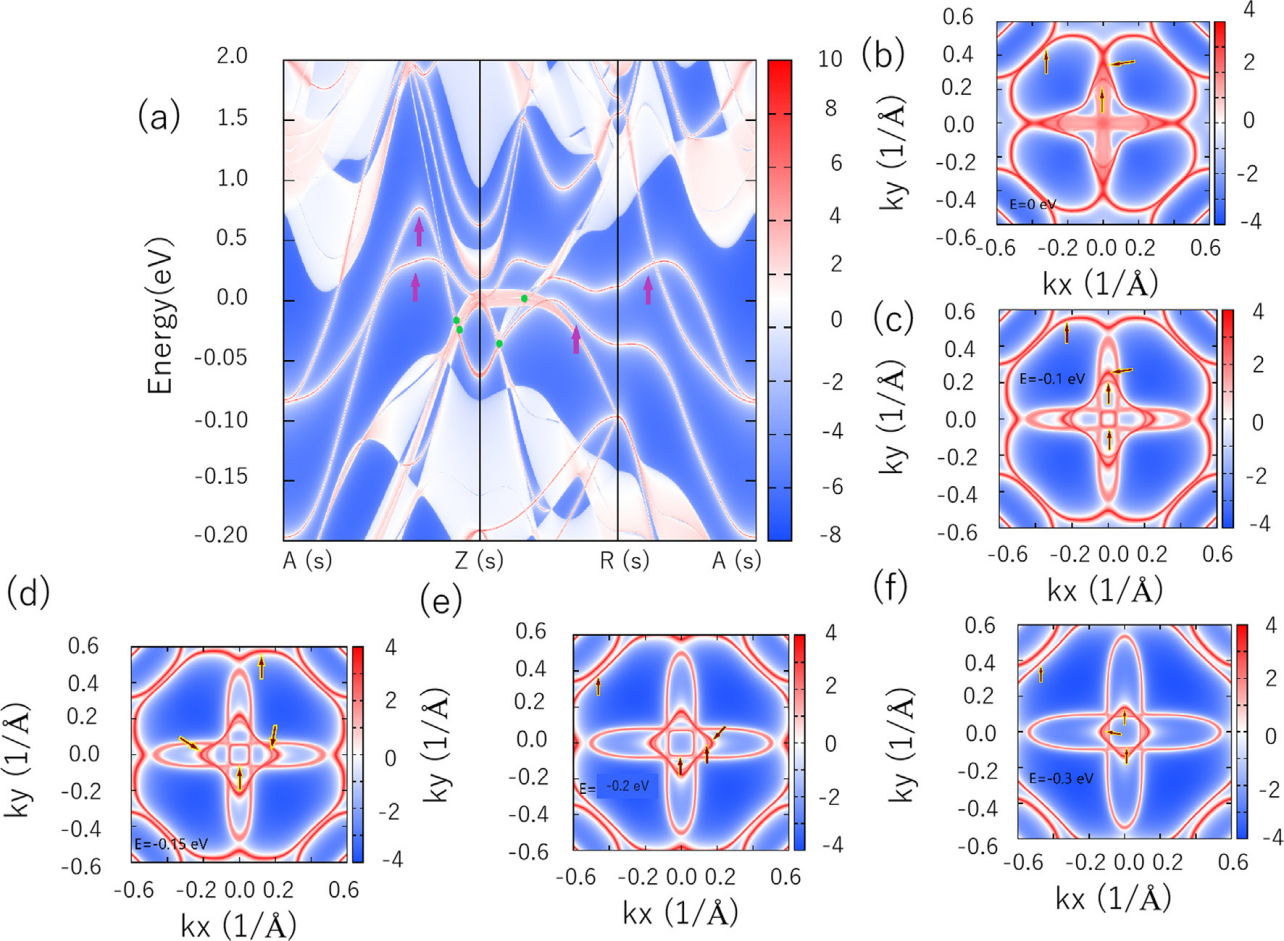
(a) Projected spectrum of the (0 0 1) surface of *anti*-PbFCl-type NaAlGe compound. Some constant energy slices at (b) *E* = 0 eV (*E*_F_); (c) −0.1 eV; (d) −0.15 eV; (e) −0.2 eV; and (f) −0.3 eV, respectively. The arrows highlight the D-H-L surface states, and the green balls indicate the location of the four BCPs.

One of the most obvious features of the TNLS is the presence of a ‘drum-head-like (D-H-L)’ surface state inside/outside the projected bulk TNLs, which can be determined via the Berry phase [52], [53], [54]. To confirm the existence of this particular D-H-L surface state, we calculated the projected spectrum and different constant energy slices of the NaAlGe (0 0 1) surface along A(s)-Z(s)-R(s)-A(s) in the surface Brillouin zone (BZ) (see Fig. S3), and the results are exhibited in Fig. 4. In Fig. 4(a), we use four green balls to indicate the location of the four BCPs and we use purple arrows to highlight the D-H-L surface states. From the figure, we can clearly see that some D-H-L surface states arise from the bulk TNLs. Different constant energy slices at *E* = 0 eV (Fig. 4(b)), *E* = −0.1 eV (Fig. 4(c)), *E* = −0.15 eV (Fig. 4(d)), *E* = −0.20 eV (Fig. 4(e)), and *E* = −0.30 eV (Fig. 4(f)) were calculated with the help of WannierTools software [43]. As we know, the NaAlGe system exhibits four TNLs (A1, A2, B1, and B2) in total, and therefore, up to four D-H-L surface states can be found in the above mentioned slices, with all these D-H-L surface states concentrated at the Z(s) high symmetry point. More importantly, as exhibited in Fig. 4, the D-H-L surface states of NaAlGe are very clear, which makes the special surface characteristics of this material very suitable for experimental observation [22], [53].

As we mentioned above, NaAlGe does not contain heavy elements, so its SOC effect is not significant. In order to further prove our viewpoint, however, we also consider the influence of the SOC on the energy structures near the *E*_F_. Figs. S4(b) and (c) show the electronic structures of NaAlGe along the M- Γ -X and A-Z-R directions, respectively. From it, we found that all CPs were opened to a certain degree of energy gap under the influence of the SOC. In region A, the SOC-induced band gaps are 3.4 meV at the maximum and 0.5 meV at the minimum; and in region B, the SOC-induced band gaps are between 0.2 meV and 2.1 meV. As shown in Fig. S4, we should point out the NaAlGe can be well described as a TNLS due to its gap sizes throughout the nodal line are less than 5 meV, which is much lower than typical TNLSs such as ZrSiS (>20 meV) [55], [56], TiB_2_ (>25 meV) [57], Mg_3_Bi_2_ (>36 meV) [58], Cu_3_PdN (>60 meV) [59], CaAgBi (>80 meV) [60].

Furthermore, the influence of biaxial strain [61] on the electronic structures of NaAlGe compound was studied. As we have shown in Fig. 2, according to the energy band that we calculated, NaAlGe is a TNLS with hybrid type NLs. A series of phase transition can be found, however, under the effect of the biaxial strain in the *ab*-plane, and the results are given in Fig. 2(c). In detail, when we applied a 2% compressive biaxial strain to the system, the slope of the crossing bands along the Γ -X direction (A1) was changed (See Fig. S6(c)). In this case, the A1 nodal line changed from type I (ground state) to hybrid type (−2%). As shown in Fig. S6(e)–(h), the energy band ordering at 3% and 4% compression biaxial strain is the same as that at 2% compression biaxial strain, so we will not analyze it in detail here. When the applied biaxial stress increases to −5%, the topological inversion characteristic of the bands along the A-Z-R direction disappear, which means that the two TNLs in region B disappear. For the two NLs in region A, the hybrid nodal line A1 still exists (see Fig. S6(i)), but the type I nodal line A2 is completely destroyed (see Fig. S6(j)). The reason can be shown as follows: band 2 moves toward the high energy level under the −5% biaxial strain, and the CPs between band 2 and band 3 disappear.

## Conclusion

In summary, we theoretically proposed that synthesized NaAlGe with *anti*-PbFCl type structure is a TNLS. It naturally exhibits four NLs, with two type I NLs at *k*_z_ = 0; the other two nodal points, one hybrid type NL (B1) and one type I NL (B2), are located in the *k*_z_ = *π* plane. All of the NLs in NaAlGe exist near the *E*_F_ and do not coexist with other bands. More importantly, the D-H-L surface states from the bulk NLs were clearly identified, which makes them well suited for experimental testing. Via biaxial strain, the size of the NLs can be actively adjusted, and different types of NLs can be observed in this system, making NaAlGe's NL features more interesting. The SOC has little effect on the energy band near the *E*_F_ in this material, which means that the NLs in NaAlGe material, which is composed of light elements, are highly resistant to SOC effects. NaAlGe was experimentally synthesized 40 years ago, but this material has not received widespread attention. Based on this work, this old compound was rejuvenated as a TNLS. It is hoped that such novel topological elements can be soon examined by experimental work.

## Compliance with Ethics Requirements

This article does not contain any studies with human or animal subjects.

## Declaration of Competing Interest

The authors declare that they have no conflicts of interest.
