# Supplementary material for: Rich topological nodal line bulk states together with drum-head-like surface states in NaAlGe with anti-PbFCl type structure
Source: J Adv Res. 2020 Jan 31;23:95–100. doi: 10.1016/j.jare.2020.01.017 (PMC7109329; doi:10.1016/j.jare.2020.01.017)
Supplement: Supplementary data 1 [file mmc1.doc]

**Supplementary Information**


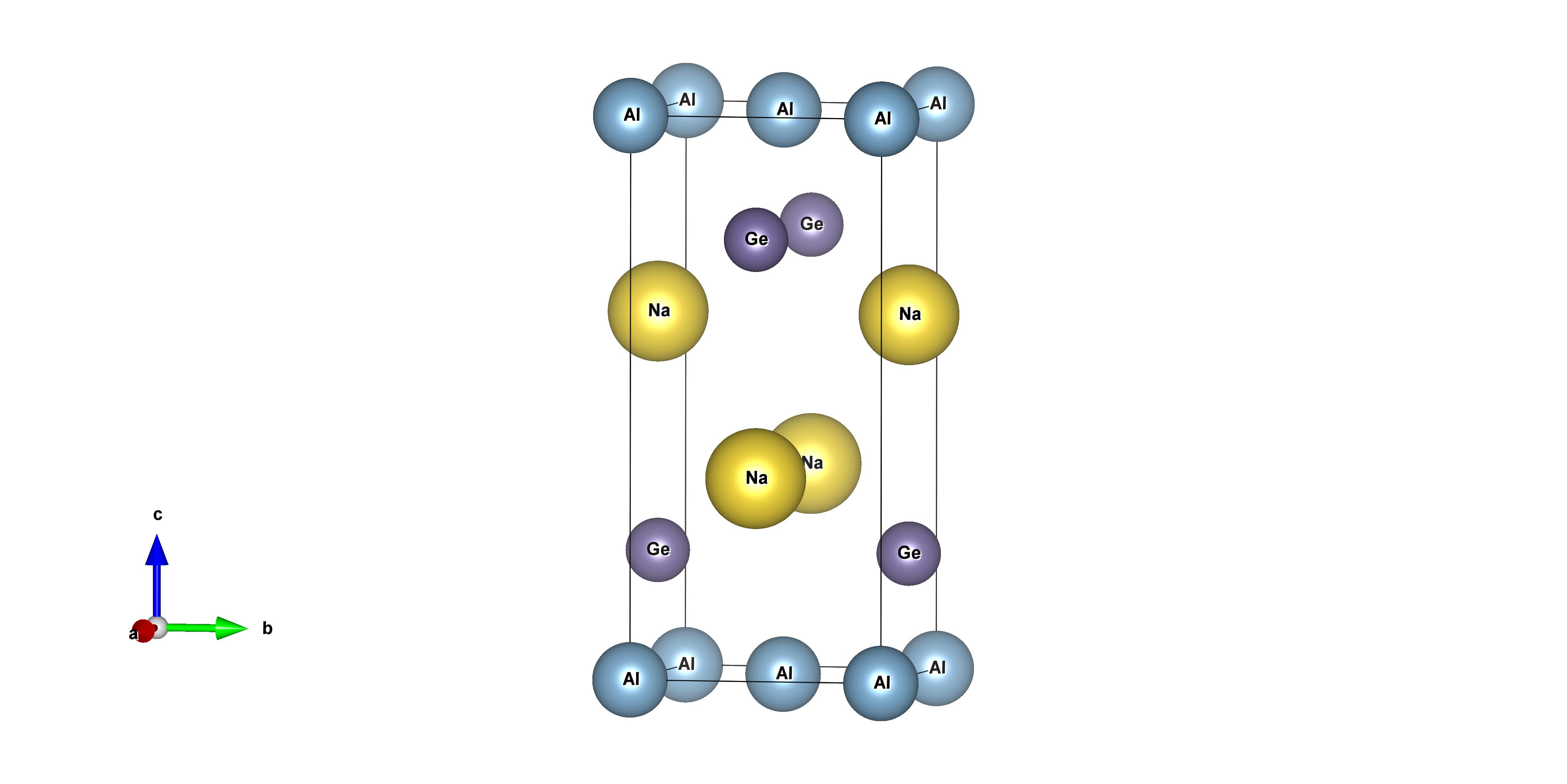

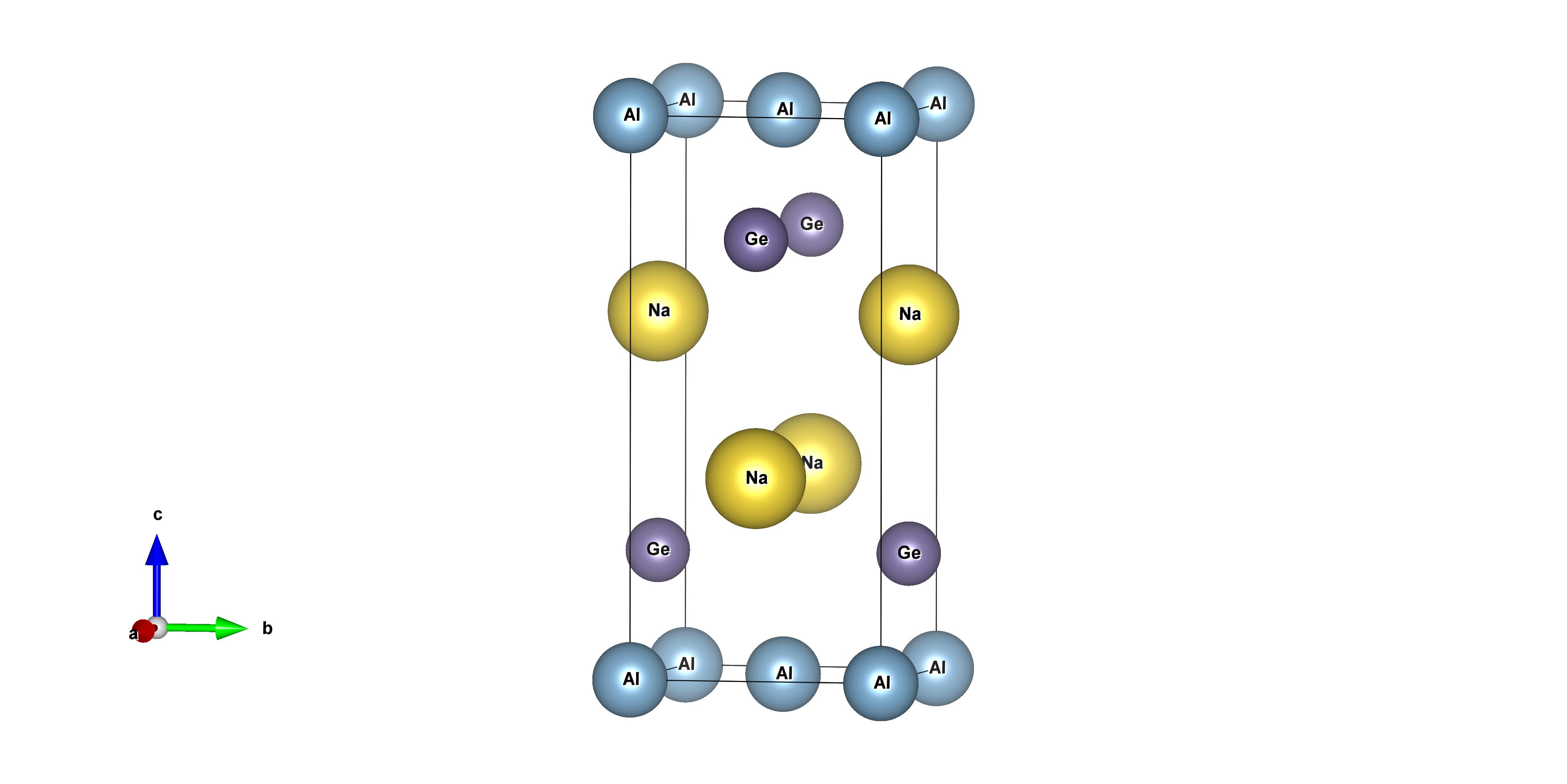


Fig. S1 Crystal structure of *anti*-PbFCl-type NaAlGe compound.


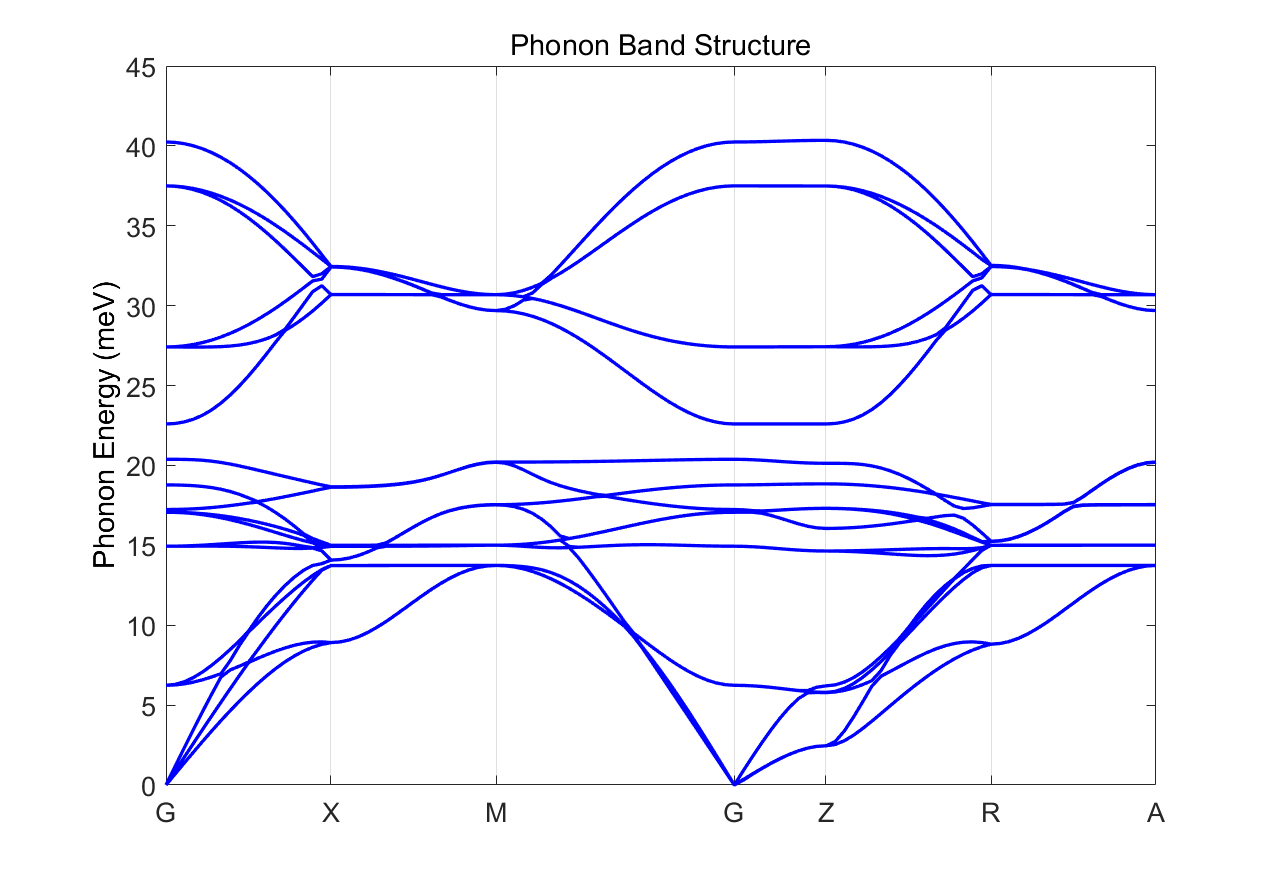


Fig. S2 Phonon band structure of *anti*-PbFCl-type NaAlGe compound.


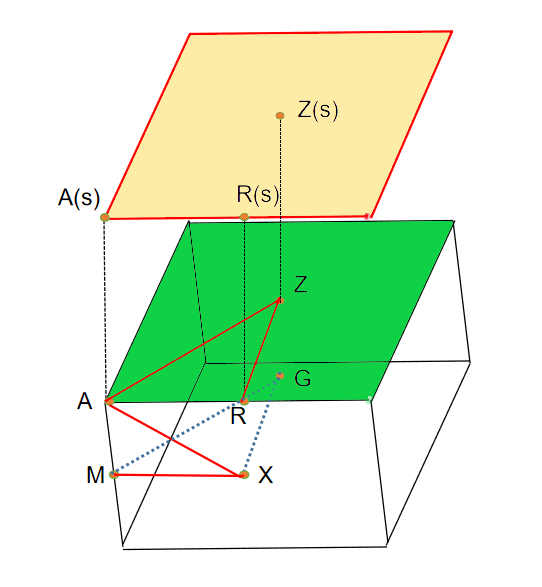


Fig. S3 Bulk Brillouin zone and its projections onto the (001) surface.


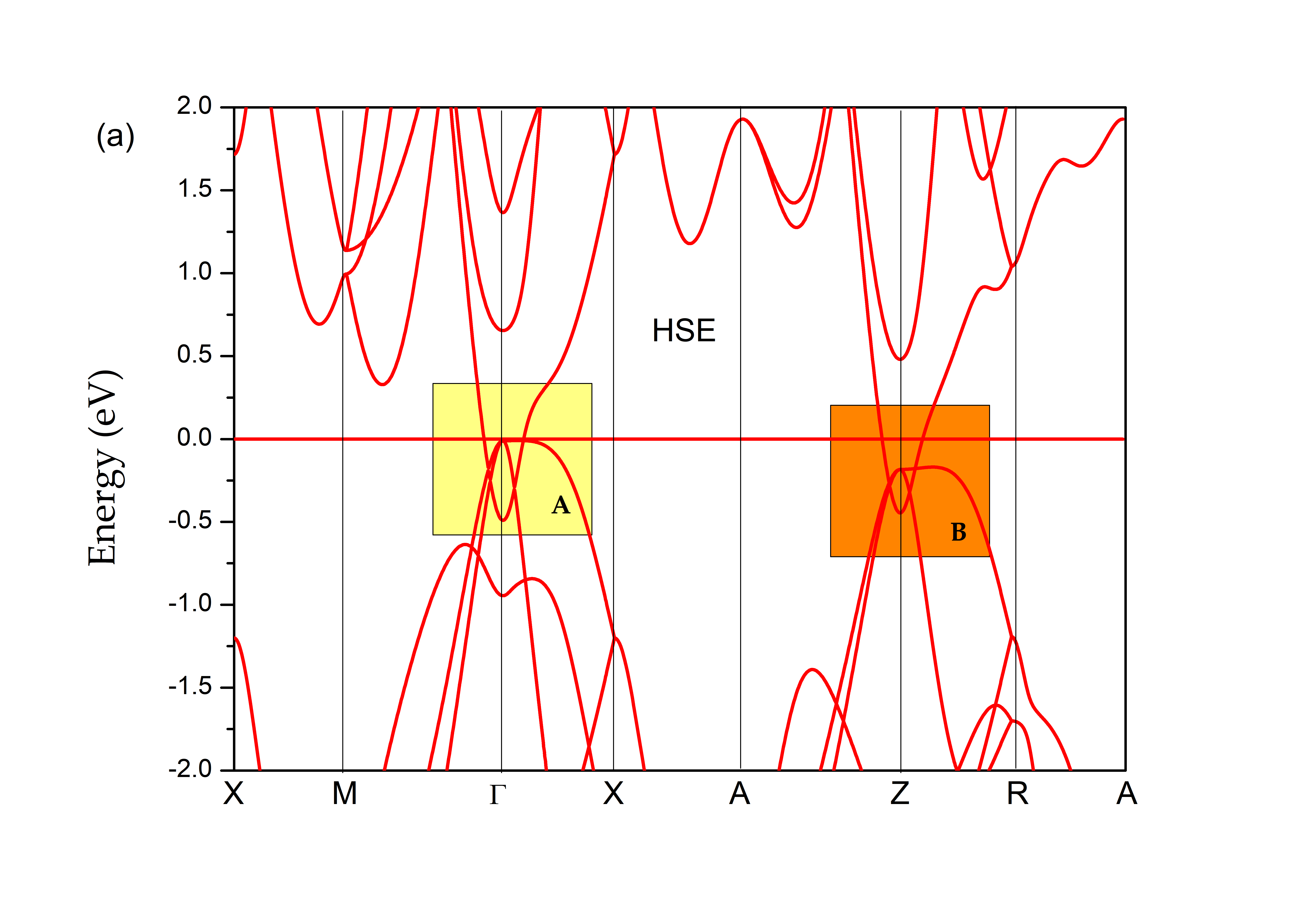


.
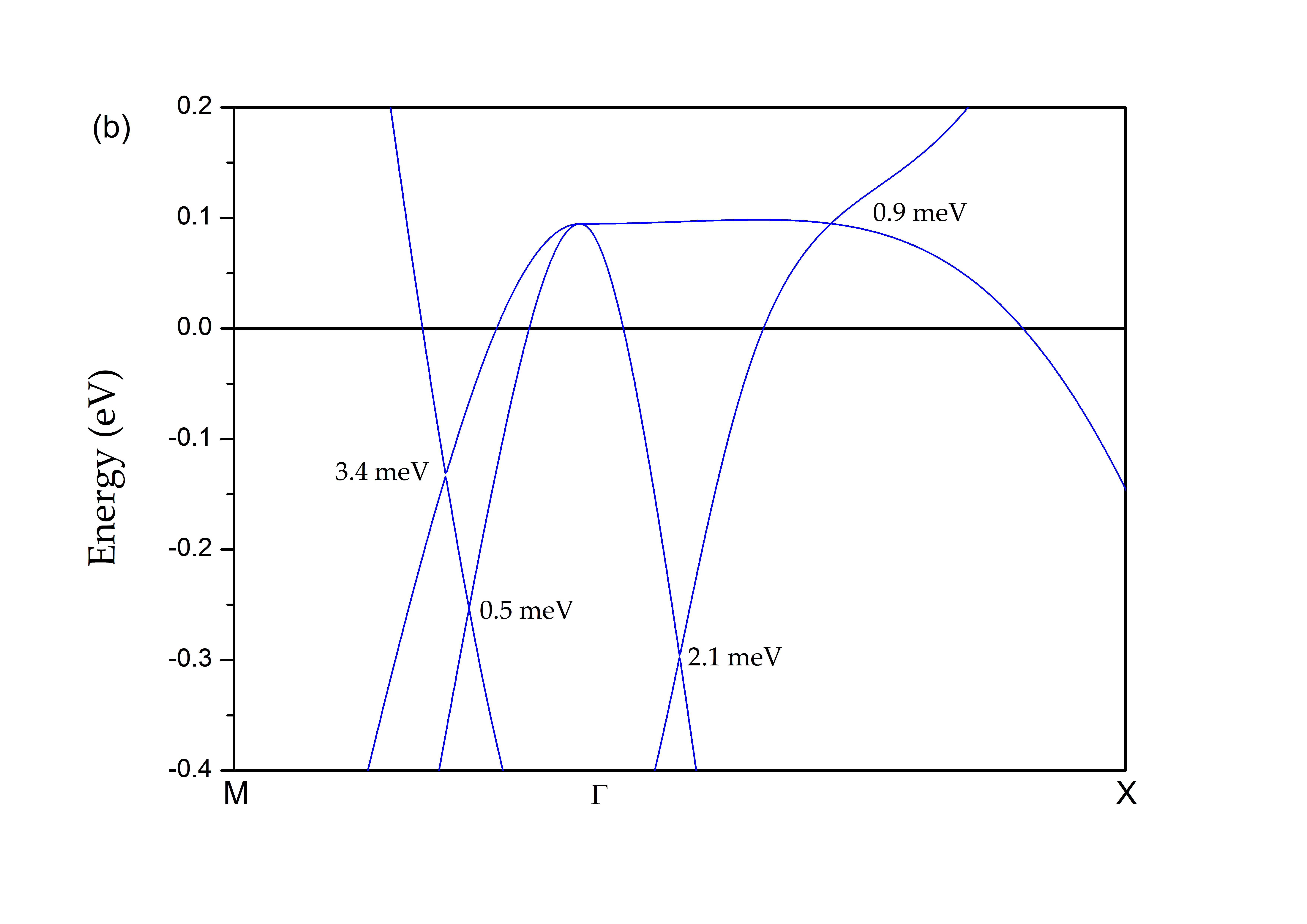


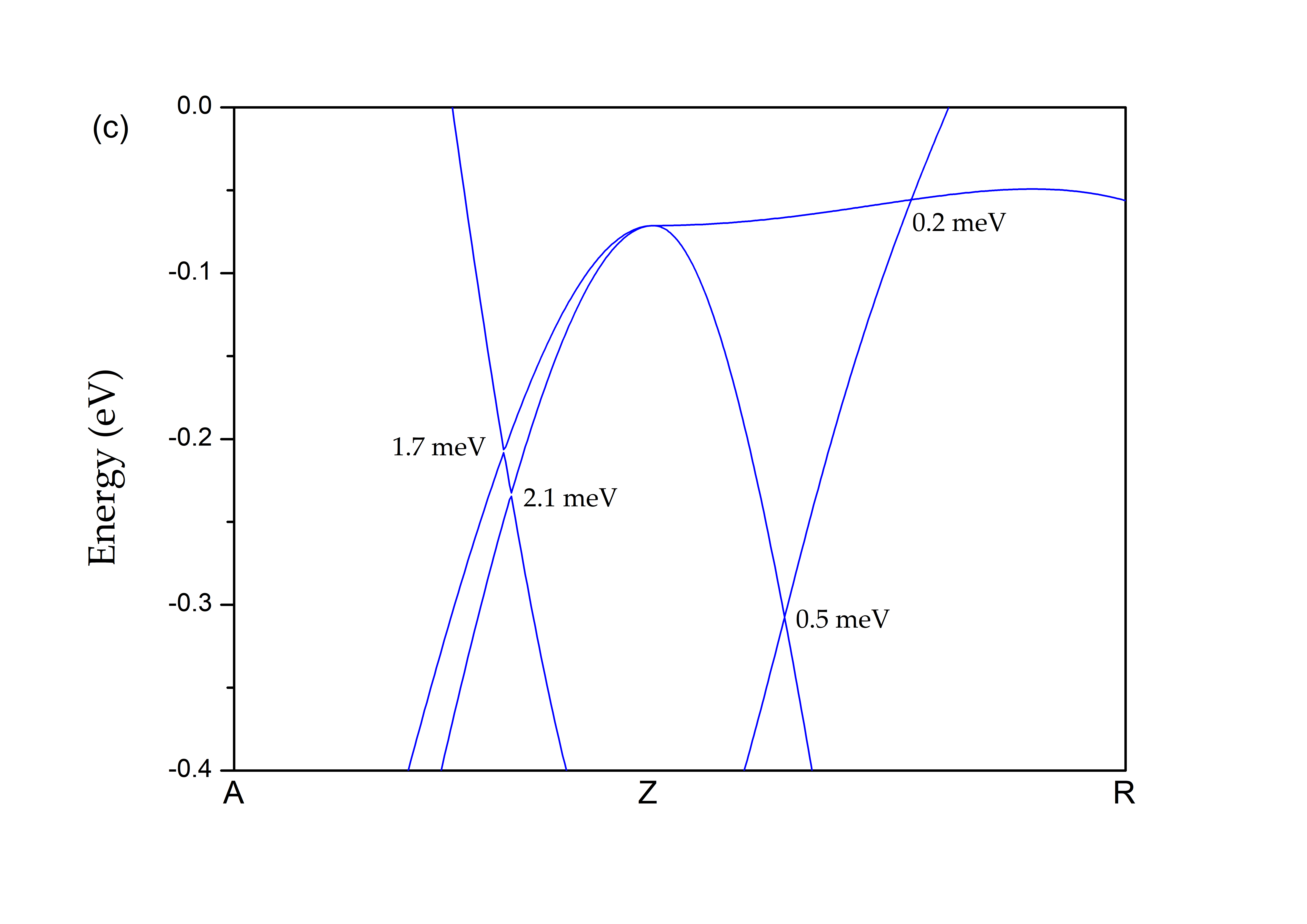


Fig. S4 Band structure of *anti*-PbFCl-type NaAlGe, calculated with the help of HSE along the high symmetry points X-M-Γ-X-A-Z-R-A in the bulk Brillouin zone; (b) and (c) Band structures of *anti*-PbFCl-type NaAlGe calculated with the help of PBE plus SOC along the high symmetry points M-Γ-X and A-Z-R, respectively, in the bulk Brillouin zone.


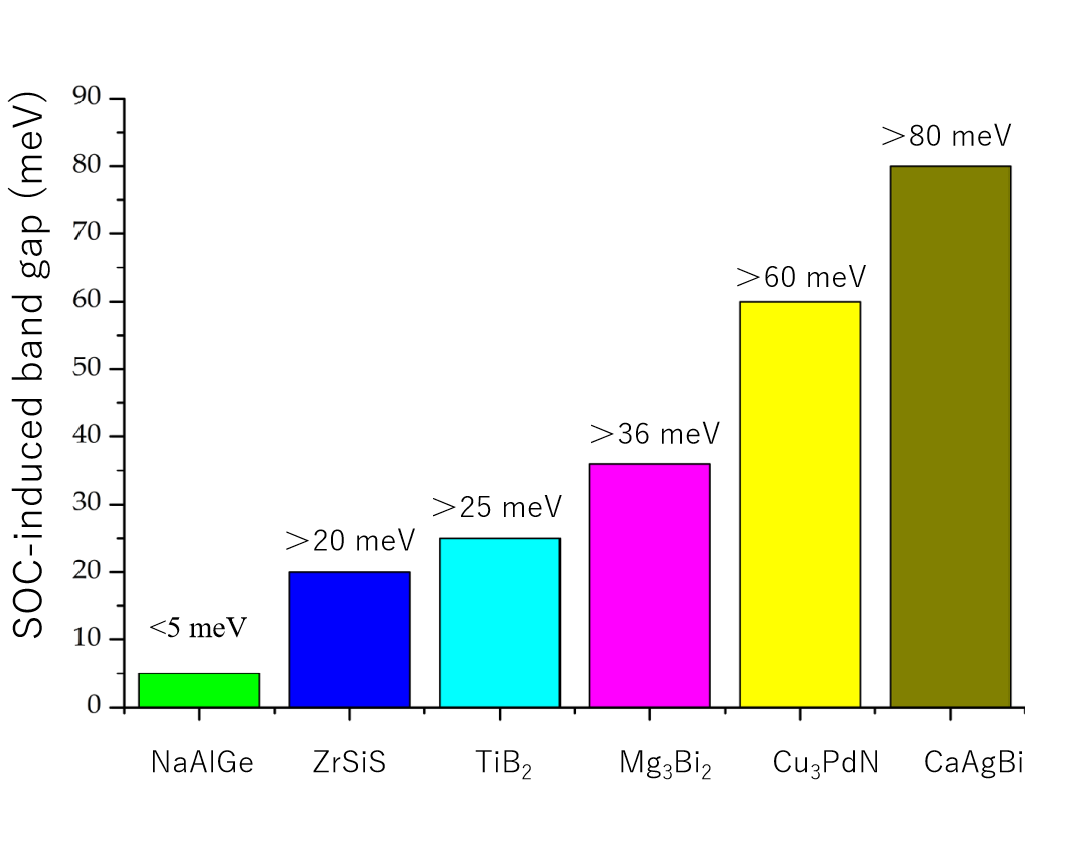


Fig. S5 SOC-induced band gaps of some well-known TNLSs.


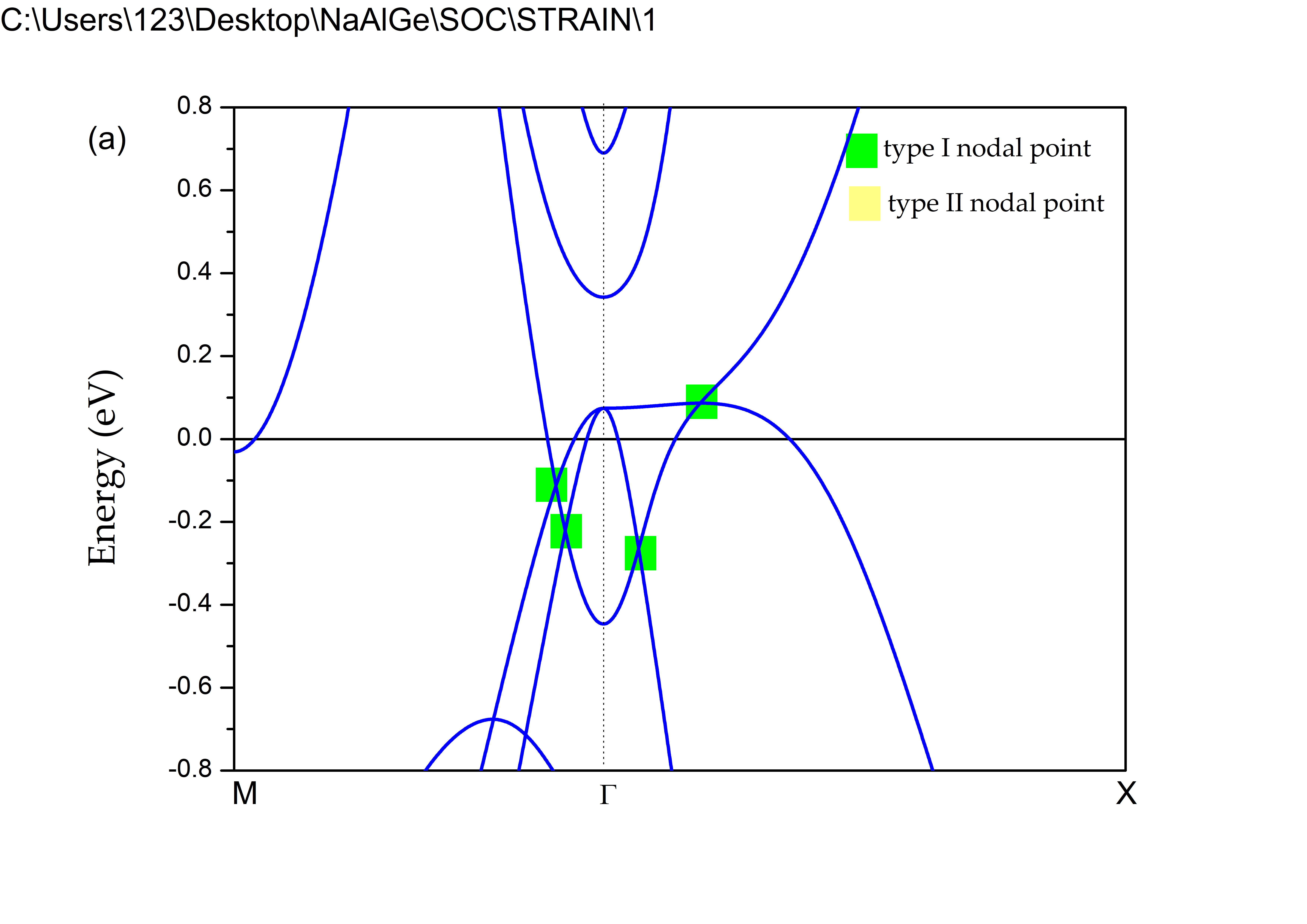

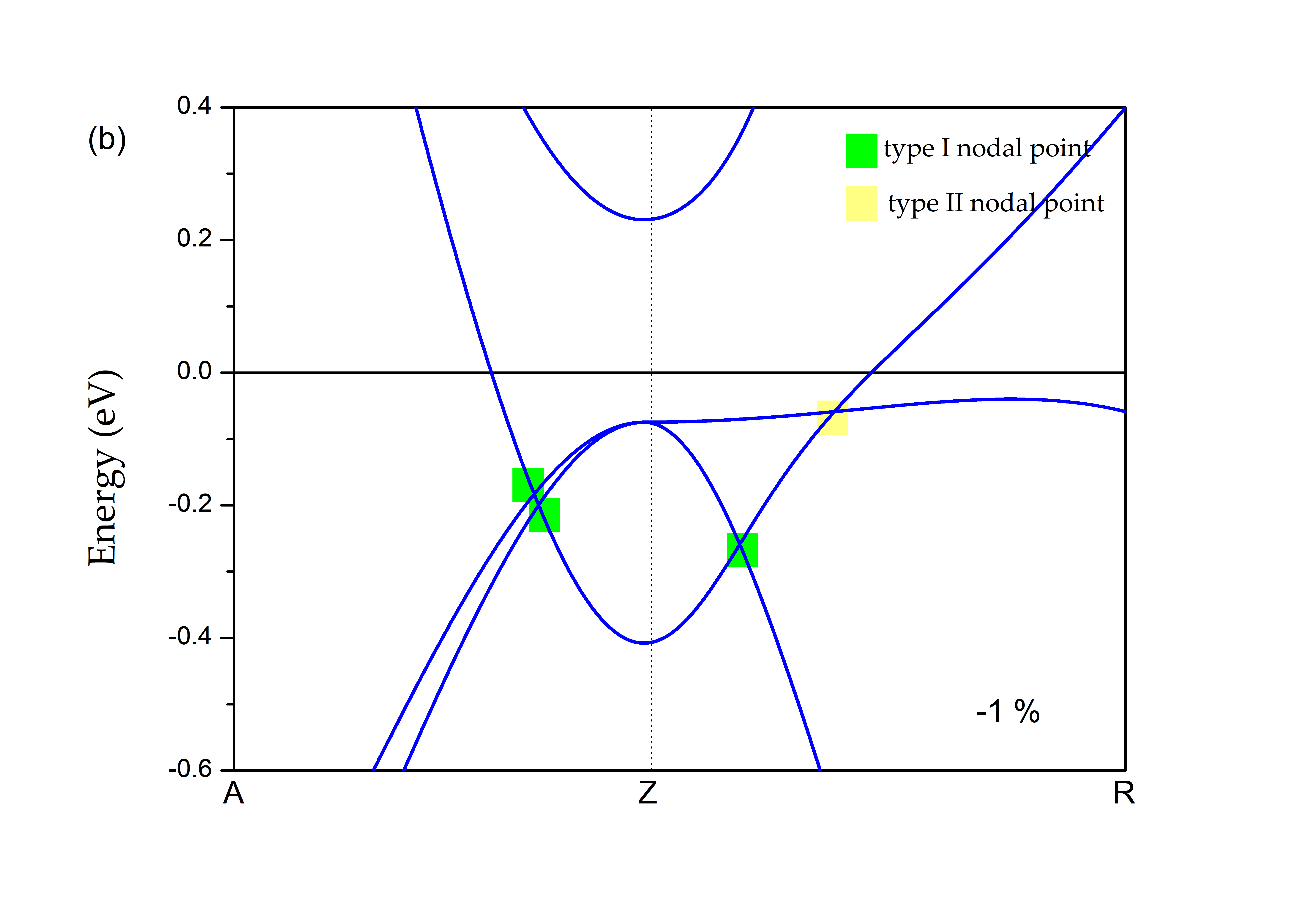

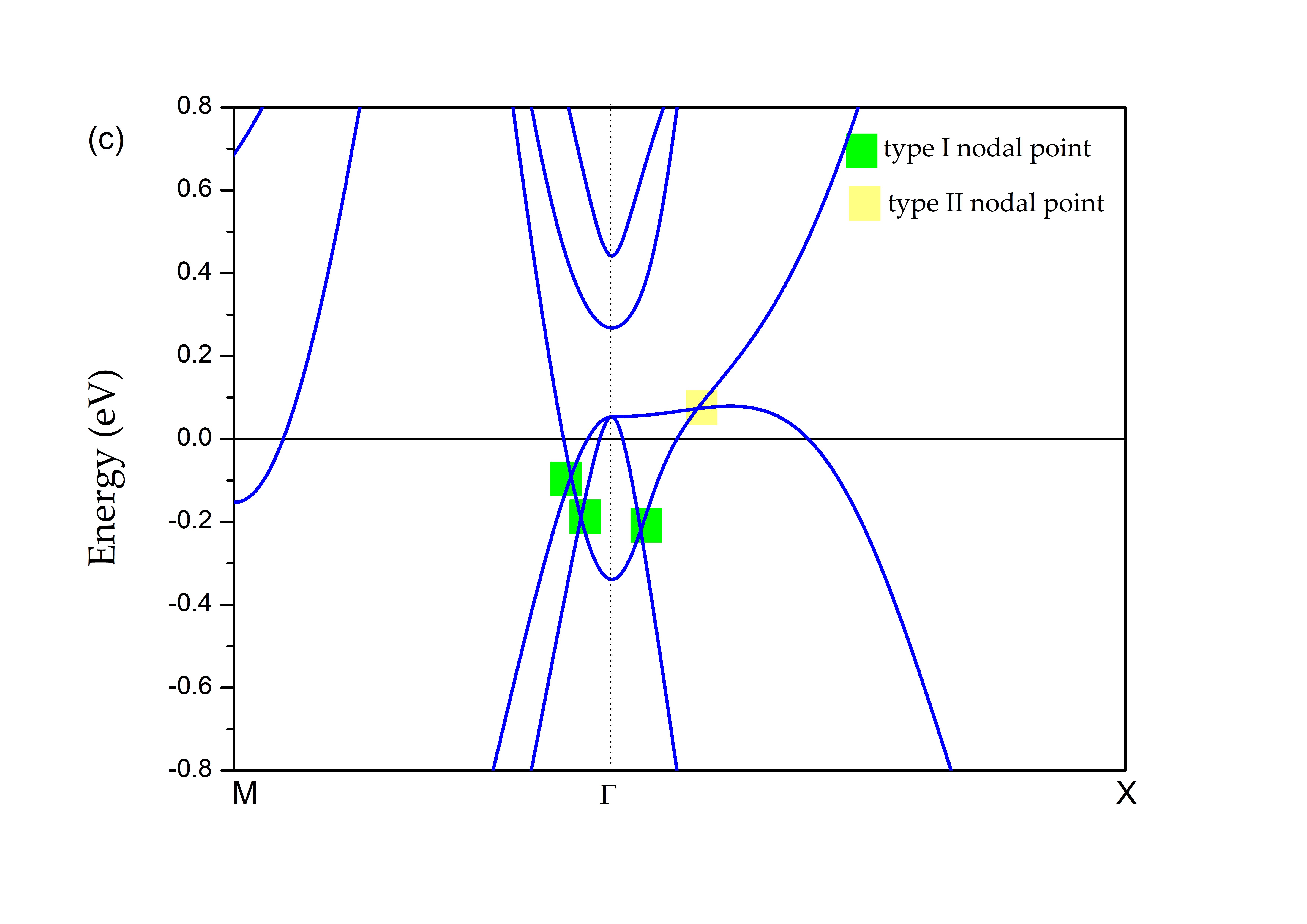

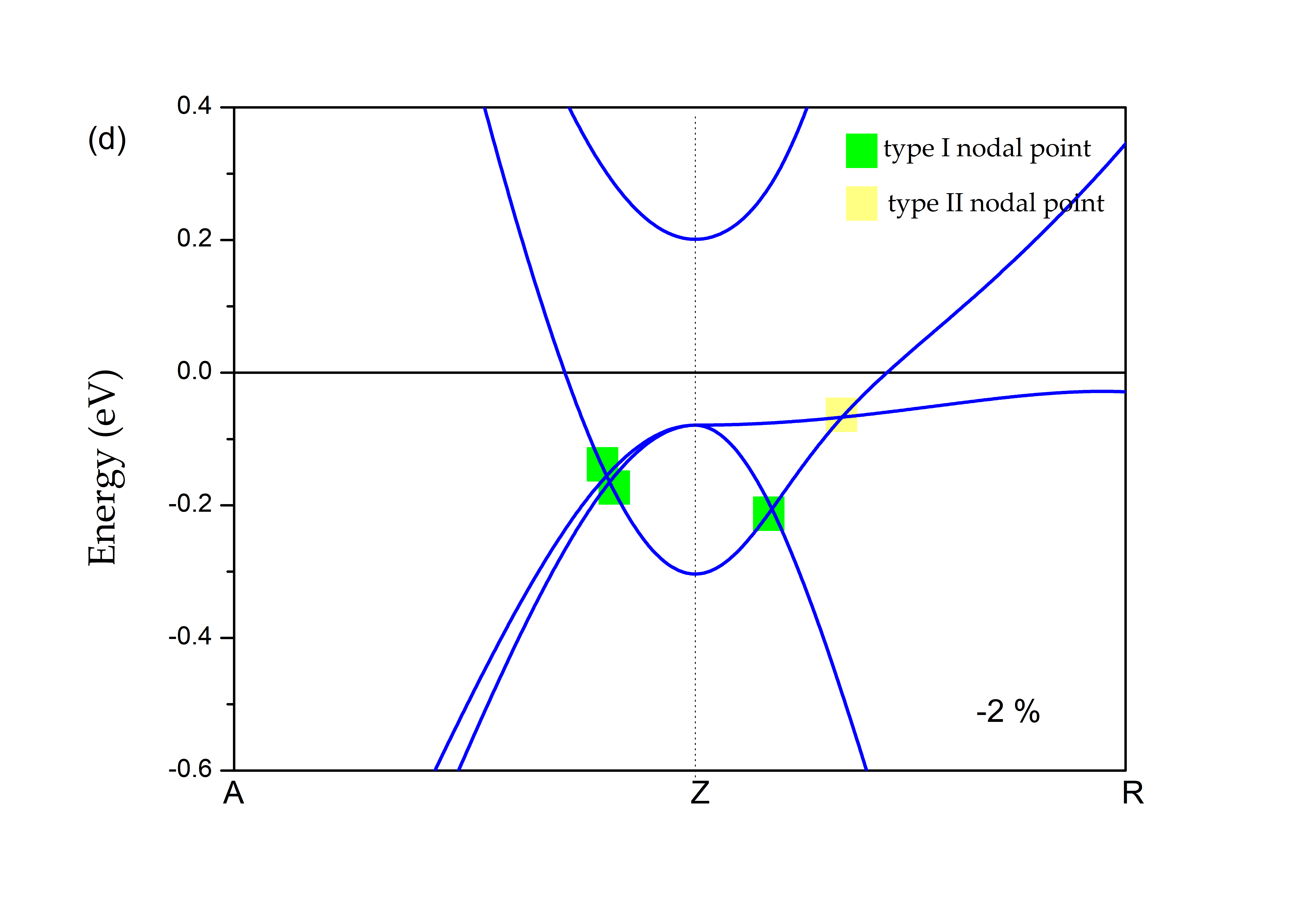

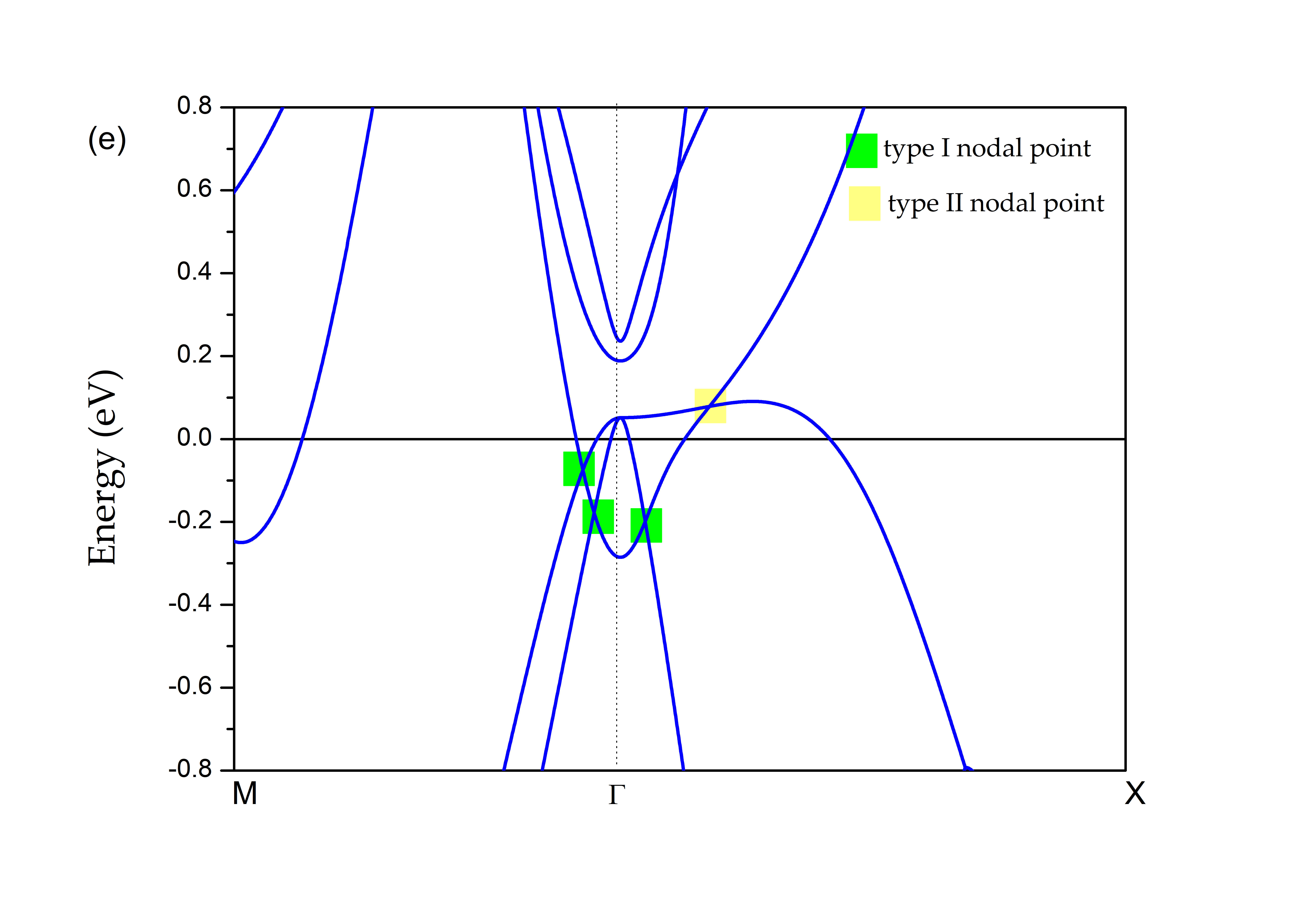

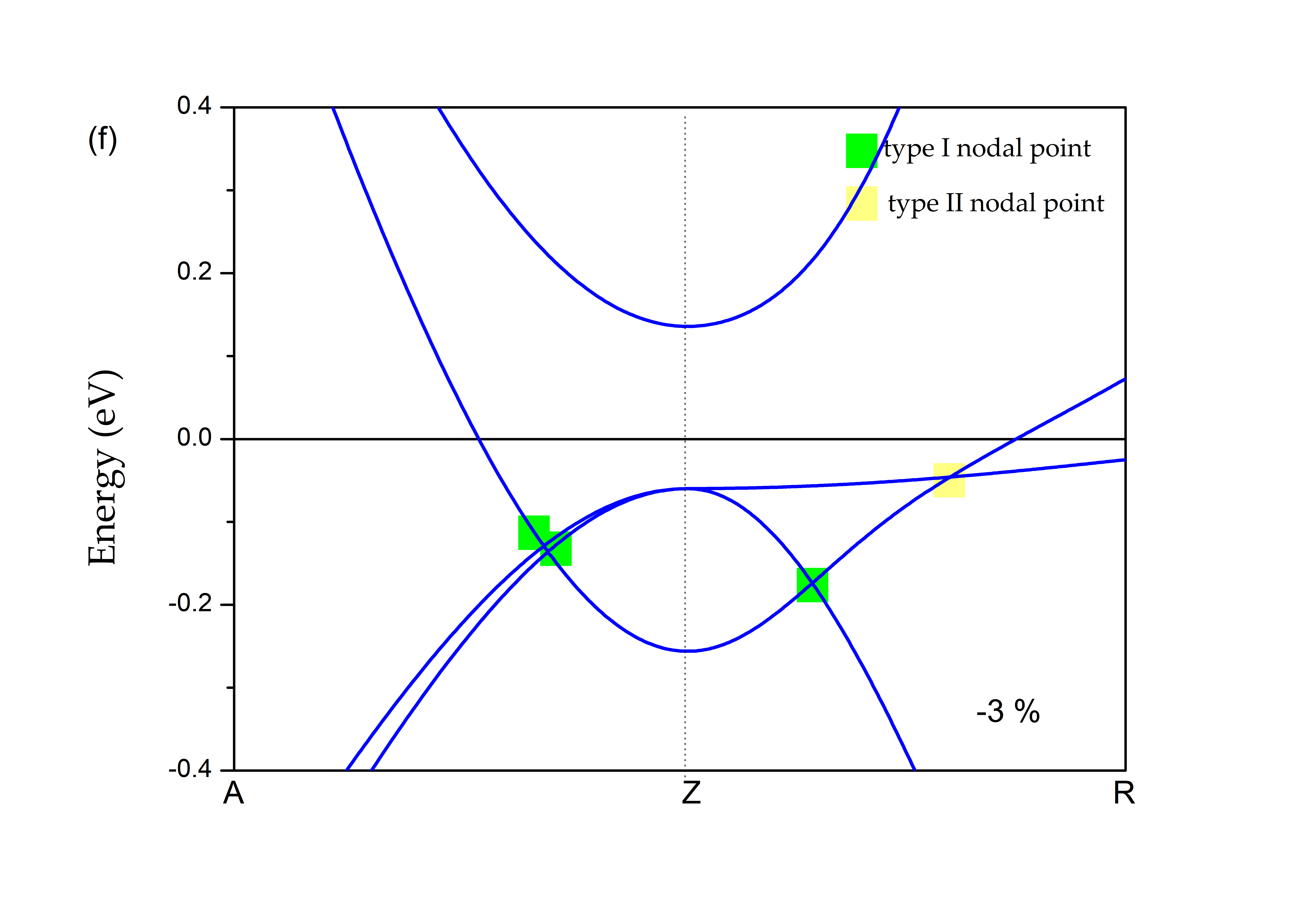


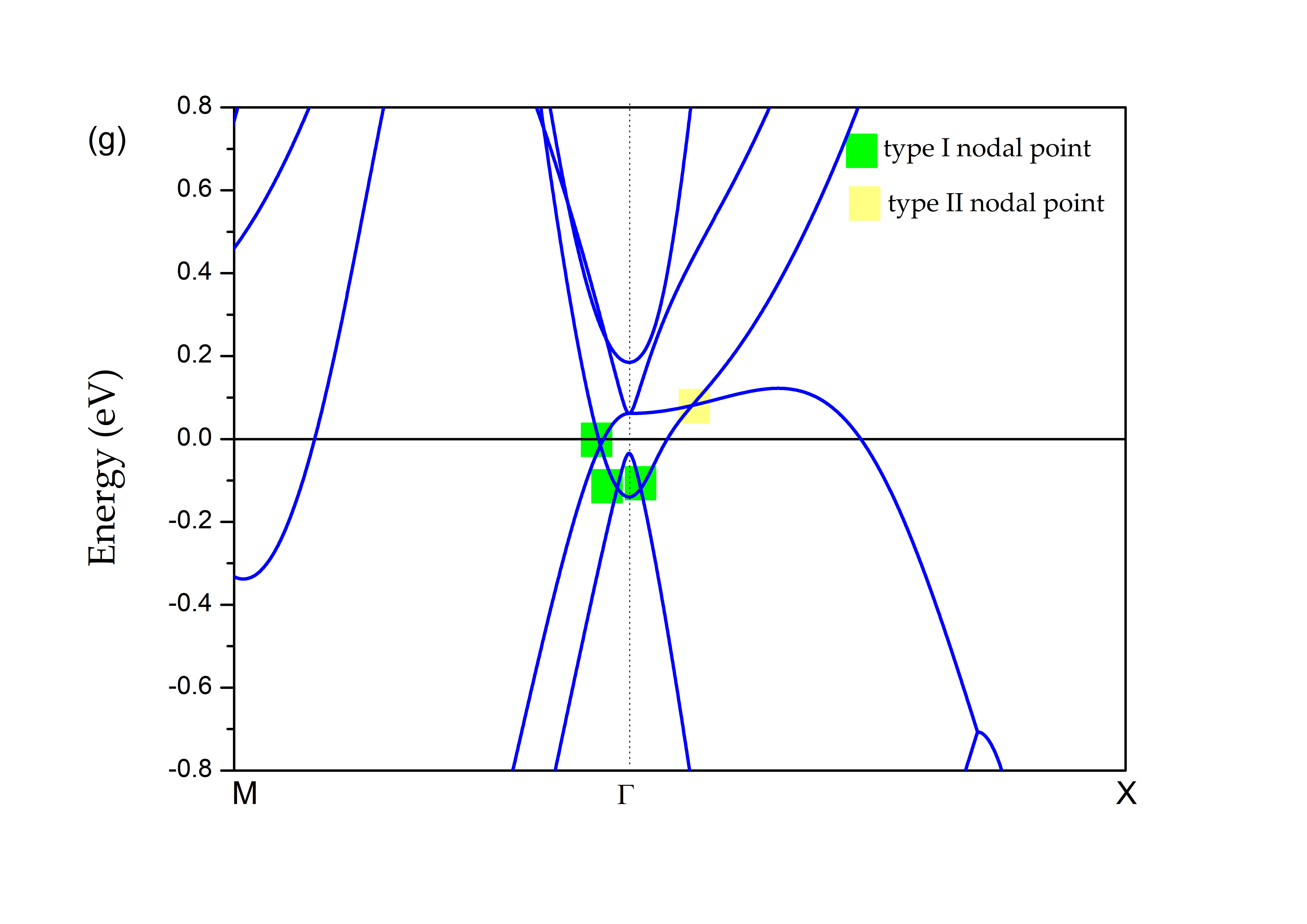

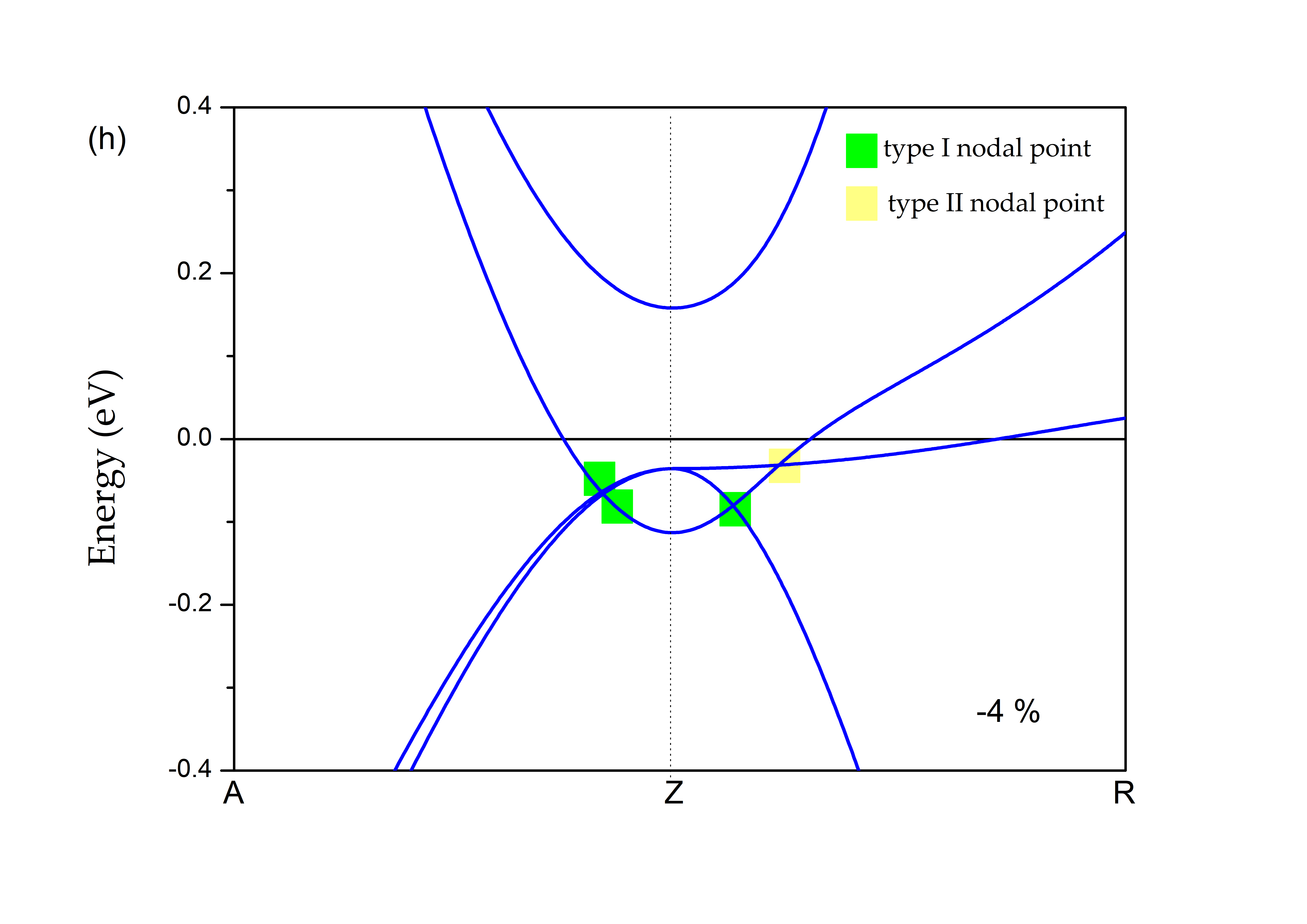

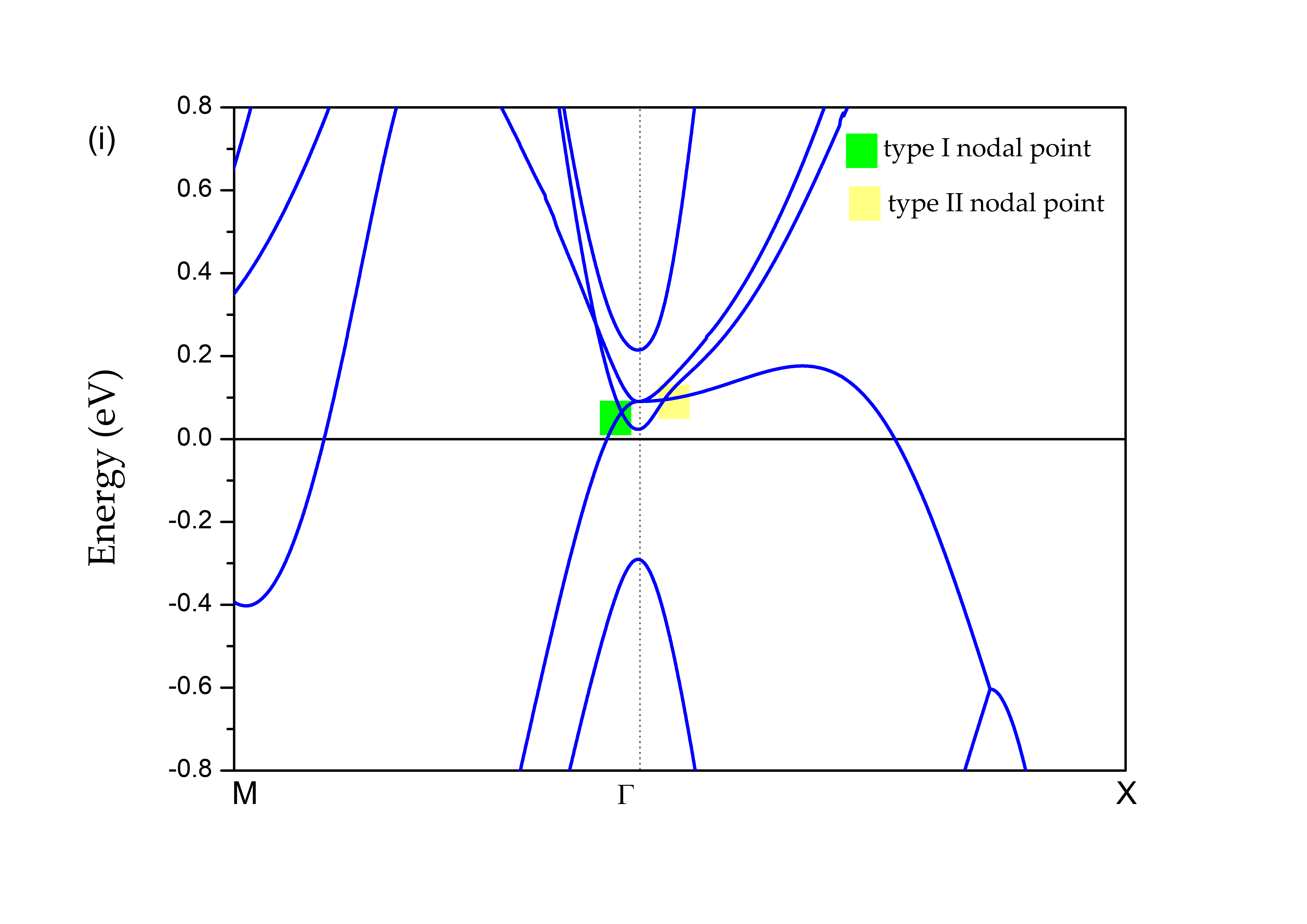

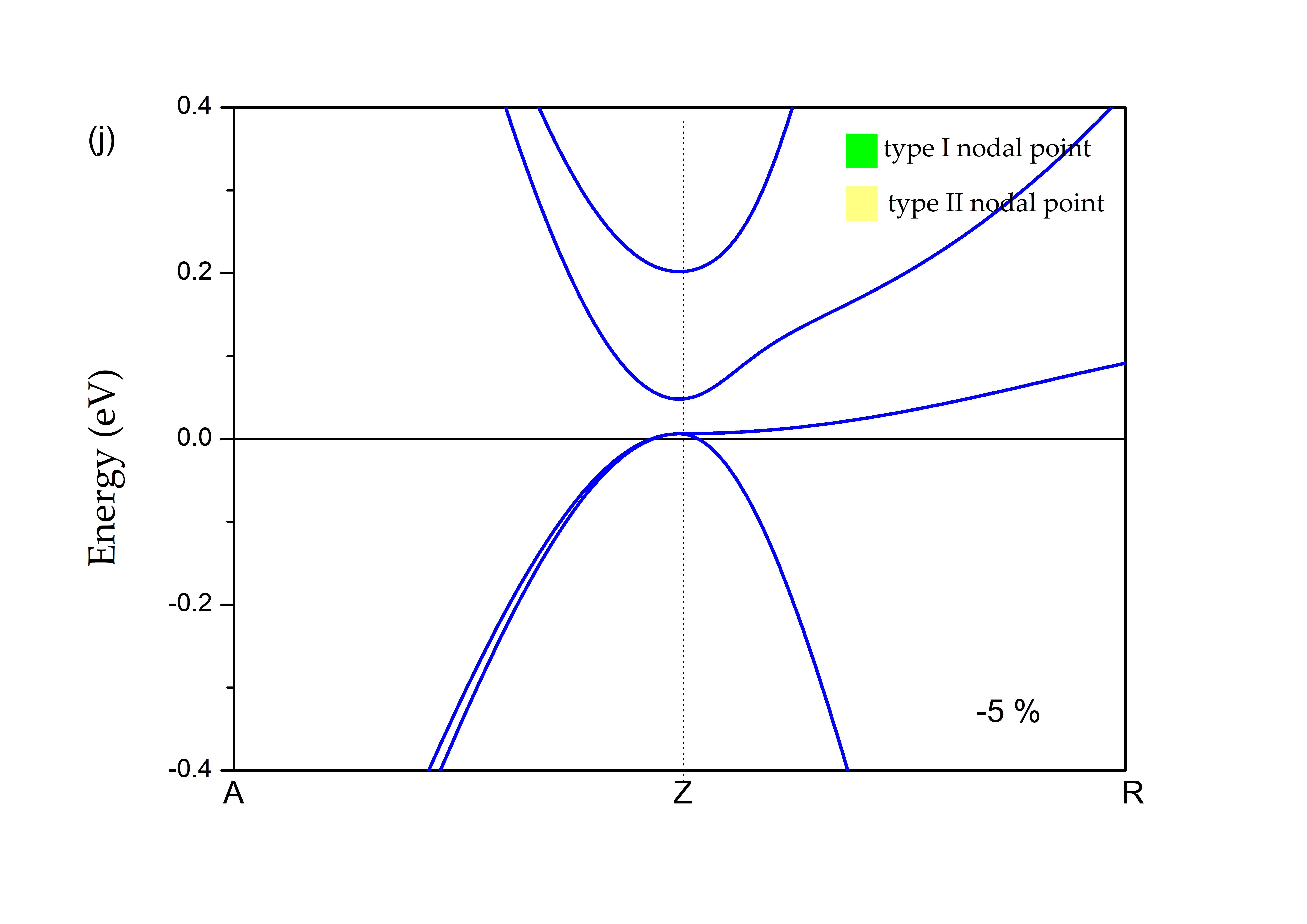


Fig. S6 Band structure of *anti*-PbFCl-type NaAlGe, calculated with the help of PBE under biaxial strain ((a, b) -1%; (c, d) -2%; (e, f) -3%; (g, h) -4%, and (i, j) -5% in the *ab*-plane along the high symmetry points M-Γ-X ((a), (c), (e), (g), and (i)), and A-Z-R ((b), (d), (f), (g), and (j)), respectively, in the bulk Brillouin zone.

E**lastic constants and mechanical properties of intermetallic compound NaAlGe**

In this section, we discuss the mechanical behavior and mechanical stability of NaAlGe material. For *anti*-PbFCl-type NaAlGe, there are six independent elastic constants, namely, *C*_11_, *C*_12_, *C*_13_, *C*_33_, *C*_44_, and *C*_66_. All these elastic constants were calculated by the stress-strain method. The obtained values for all constants are reported in Table S1:

Table S1: The calculated elastic constants (*C*_ij_) for NaAlGe compound.

| Compound | *C*_11_  [GPa] | *C*_12_  [GPa] | *C*_13_  [GPa] | *C*_33_  [GPa] | *C*_44_  [GPa] | *C*_66_  [GPa] |
| --- | --- | --- | --- | --- | --- | --- |
| NaAlGe | 65.76 | 42.02 | 16.43 | 58.03 | 16.91 | 20.40 |

The mechanical stability of this system can be evaluated by the following necessary and sufficient conditions:

Criteria (i) *C*_11_ > |*C*_12_| (met);

Criteria (ii) 2×*C*_13_^2^ < *C*_33_ (*C*_11_ + *C*_12_) (met);

Criteria (iii) *C*_44_ > 0 (met).

From the various calculated elastic constants, it is confirmed that NaAlGe compound fulfills all the conditions and thus is mechanically stable, as its experimental synthesis has already been confirmed and reported. The average mechanical properties for NaAlGe are also given in Table S2. We hope that the theoretical data that we have obtained here can provide a reference for subsequent experimental work.

Table S2: The calculated average mechanical properties for NaAlGe.

| Scheme | Voigt | Reuss | Hill |
| --- | --- | --- | --- |
| Bulk modulus K (GPa) | 37.704 | 36.143 | 36.923 |
| Shear modulus G (GPa) | 19.192 | 17.798 | 18.495 |
| Young's modulus E (GPa) | 49.223 | 45.866 | 47.546 |
| P-wave modulus (GPa) | 63.293 | 59.874 | 61.583 |
| Poisson's ratio v | 0.282 | 0.288 | 0.285 |
| Bulk/Shear ratio | 1.965 | 2.031 | 1.996 |
